# Factor structure and psychometric properties of the Perceived Stress Scale in Russian adolescents

**DOI:** 10.1038/s41598-023-51104-1

**Published:** 2024-01-08

**Authors:** Julia Marakshina, Timofey Adamovich, Georgy Vasin, Victoria Ismatullina, Marina Lobaskova, Artem Malykh, Pavel Kolyasnikov, Anna Tabueva, Ilya Zakharov, Sergey Malykh

**Affiliations:** 1 Center of Population Research, Ural Institute of Humanities, Ural Federal University Named After the First President of Russia B.N. Yeltsin, Yekaterinburg, Russia; 2 Yerevan, Armenia; 3 Developmental Behavioral Genetics Lab, Federal Research Centre of Psychological and Interdisciplinary Studies, Moscow, Russia

**Keywords:** Psychology, Human behaviour

## Abstract

This study involved a psychometric analysis of the 10-item Perceived Stress Scale (PSS-10). To investigate the Russian version of the PSS-10 for adolescents, 3530 adolescents aged 13–17 years were recruited. Confirmatory factor analysis revealed that the data corresponded to the expected two-factor configuration. Psychometric properties and factor structure were evaluated. As expected, the PSS-10 included two factors: perceived helplessness and perceived self-efficacy. Internal consistency demonstrated acceptable values (Cronbach’s alpha was 0.82 for perceived helplessness, 0.77 for perceived self-efficacy, and 0.80 for the overall PSS score). Measurement invariance across sexes was assessed, and configural and metric invariance were confirmed. The developed diagnostic tool can be used both in the school system to alleviate the negative consequences of academic stress in adolescents and, in the future, in other areas, particularly in clinical practice.

## Introduction

Stress is often linked to harmful or dangerous events, which can cause unpleasant feelings and moods^1^. However, stress is also a natural human response to both internal and external threats^2^. Perceived stress is the result of an individual's assessment of the stressor (as threatening or nonthreatening), along with their ability to cope with it. Accurately assessing stress levels is important for developing effective strategies to manage stress. Stress causes a nonspecific physiological reaction^3^, and in psychological and physiological research, this is typically referred to as distress^4^. The physiological effects of stress are associated with the functioning of the autonomic nervous system and the hypothalamic‒pituitary‒adrenal axis^5^. Chronic stress negatively affects the immune system, increasing the likelihood of infections and chronic diseases and increasing the risks of cardiovascular disease, depression, drug addiction, and other conditions^6–9^. A number of studies have found that stress can also affect epigenetic mechanisms, such as DNA methylation (e.g.,^10,11^). In addition, stress can impact psychological characteristics, including learning and self-control^12,13^.

Adolescents are not immune to stress; moreover, they are particularly susceptible to academic stress. The COVID-19 pandemic resulted in a transition to remote learning, which has led to an increase in stress levels in schoolchildren, as demonstrated by various studies. A review of 72 studies showed the impact of remote learning (caused by factors including the COVID-19 pandemic) on children's psychological well-being as well as emotional (e.g., stress, anxiety) and behavioral problems^14^. Remote learning is a specific circumstance that creates additional stress, as demonstrated in a number of studies, mainly in adult (student and teacher) samples^15–17^. Diagnostic tools to measure stress and coping strategies in adolescent schoolchildren are needed necessary. Even under normal circumstances, academic stress can occur, especially in adolescents with object and test anxiety. High levels of cortisol, a stress hormone, are observed in students with poor academic performance in math, and a lack of coping strategies is associated with an increase in cortisol levels during knowledge testing situations^18^. An increase in the level of cortisol predicts a decrease in the rates of successfully solving problems involving numerical skills^19^. Math anxiety is a specific form of stress that affects learning success^20^. Remote learning has also introduced new stressors that impact learning success^14,16^. Researchers have found that stress, combined with feelings of inferiority and low self-esteem, contribute to academic failure among children with learning difficulties. Effective interventions are needed to reduce the influence of stressors and increase academic success^21^. Cross-cultural differences in stress manifestations among adolescents have been identified, especially in certain groups of stressors. For instance, Chinese adolescents are more susceptible to academic achievement-related stress^22^. In recent years, Russian schools have reported an increase in bullying cases, causing additional stress among school children^23^. Special questionnaires have been developed to assess the school environment, coping strategies, and manifestations of stress^24–26^. In such situations, it is crucial to evaluate not only the psychological climate of the school but also the stress manifestations and coping strategies employed by adolescents. However, a stress questionnaire specifically designed for adolescent groups has not yet been developed or adapted.

One of the well-established scales aimed at assessing stress is the Perceived Stress Scale (PSS). The PSS was designed to measure respondents' feelings of lack of control and predictability as well as being overburdened in their lives. The Perceived Stress Scale was first proposed by Cohen et al.^27^ and originally included 14 items. The PSS-14 has been validated in various countries and populations, including adults^28–30^, women with postnatal distress^31^, city dwellers^32^, working adults^33^, relatives of suicide survivors^34^, female police officers^35^, patients with cardiovascular disease^36^, elderly service workers^37^, asthma patients^38^, patients with chronic disease^39^, postpartum women with and without preeclampsia^40^ (Torres-Lagunas et al., 2015), dementia patients^41^, university students^42^, and nursing professionals^43^. Additionally, the PSS-14 has been validated for use in adolescents^44–46^. A brief version of the PSS, which is a 4-item adaptation, has also been developed^47^. Based on the PSS-14, a 10-item adaptation was developed by Cohen^48^ and has become common for use with adult respondents across countries and populations^28,29,49–51^. The PSS-10 was adapted into Russian by Ababkov et al.^52^, and it has a two-factor structure: perceived helplessness and perceived self-efficacy. The total score on the PSS-10 is also calculated.

However, validation of use of the PSS-10 in adolescents is limited, with only one known version assessed in Chinese adolescents^22^. The Russian version has not been validated for use with adolescents. Thus, the purpose of this study was to adapt the Russian version of the PSS-10 for use in adolescents and to evaluate its internal consistency, factor structure, external validity, and sex invariance.

## Results

### Confirmatory Factor Analysis (CFA)

CFA was conducted to confirm the two-factor structure of the questionnaire. The fit indices of the model indicated good fit to the data, supporting the conclusion that the two-factor configuration of the questionnaire was appropriate. The CFA results are presented in Table 1.

**Table 1:** Fit indices of the two-factor model: Results of confirmatory factor analysis.

Index	Value
Comparative fit index (CFI)	0.986
Tucker‒Lewis index (TLI)	0.981
Root mean square error of approximation (RMSEA)	0.075
RMSEA: 90% CI (lower bound)	0.071
RMSEA: 90% CI (upper bound)	0.080
Standardized root mean square residual (SRMR)	0.045

**Figure 1:**
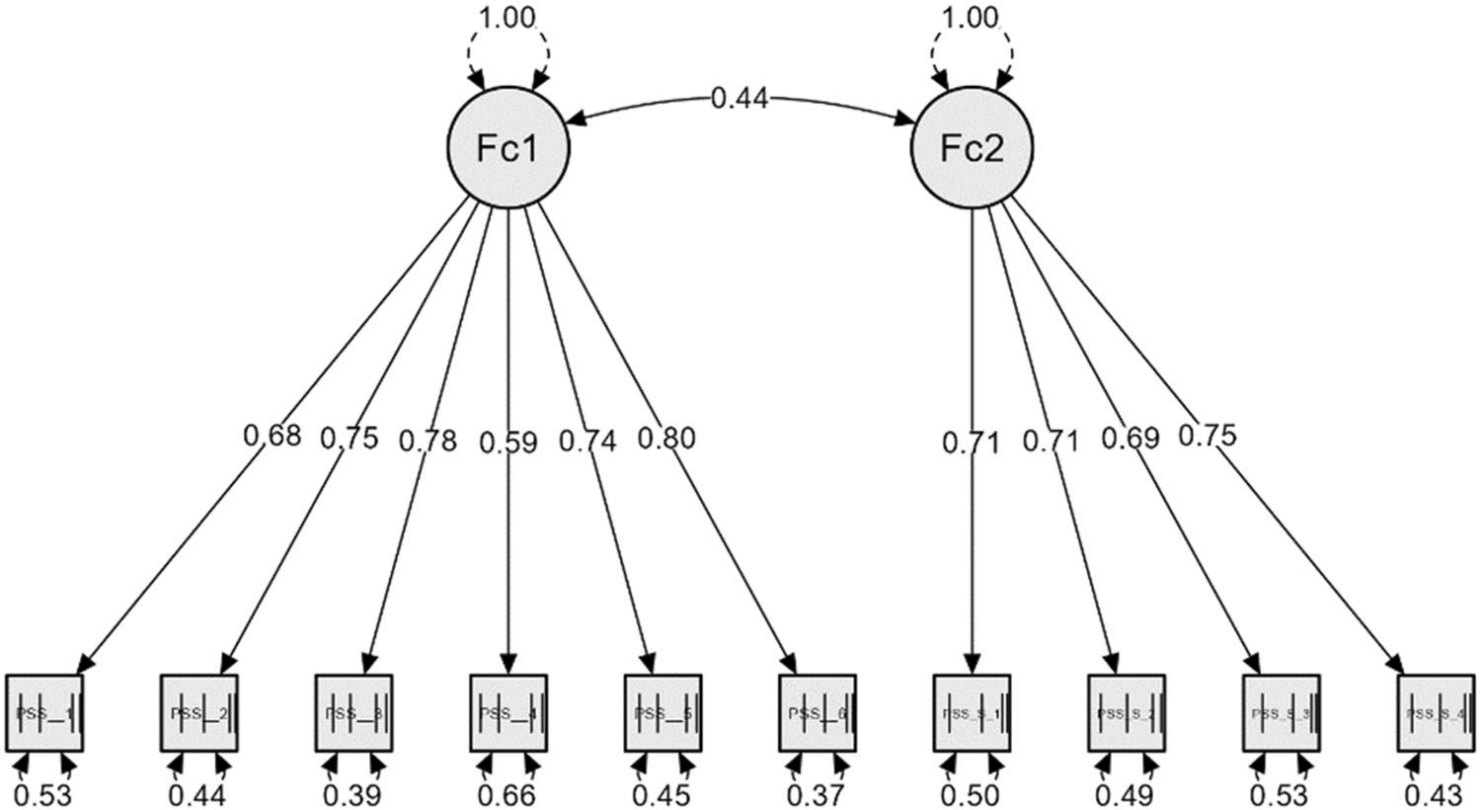
Factor structure of the Perceived Stress Scale. Fc1: Factor 1 (perceived helplessness); Fc2: Factor 2 (perceived self-efficacy).

Based on the CFA results, we defined two scales: perceived helplessness (Items 1, 2, 3, 6, 9, 10) and perceived self-efficacy (Items 4, 5, 7, 8). Factor structure of PSS is presented on Fig. 1.

### Reliability (Internal Consistency)

Regarding internal consistency, Cronbach's α values were calculated. The perceived helplessness subscale had a Cronbach’s α of 0.82, the perceived self-efficacy subscale had a Cronbach’s α of 0.77, and the PSS total score had a Cronbach’s α of 0.80. These values indicate high reliability for the questionnaire subscales.

### Descriptive Statistics

Table 2 presents descriptive statistics for the PSS subscales, including the mean and standard deviation.

**Table 2:** Descriptive statistics and reliability of the PSS scores across age groups from 13–17.

	Perceived helplessness					Perceived self-efficacy					PSS total				
Age	13	14	15	16	17	13	14	15	16	17	13	14	15	16	17
Valid	65	846	1112	920	525	65	846	1112	920	525	65	846	1112	920	525
Mean	2.54	2.47	2.52	2.48	2.57	1.36	1.45	1.40	1.32	1.32	3.91	3.92	3.92	3.80	3.89
Std. Deviation	0.74	0.76	0.77	0.82	0.83	0.81	0.78	0.75	0.77	0.79	1.23	1.20	1.22	1.32	1.39
Minimum	1.00	1.00	1.00	1.00	1.00	0.00	0.00	0.00	0.00	0.00	1.25	1.00	1.00	1.00	1.00
Maximum	4.67	5.00	5.00	5.00	5.00	4.00	4.00	4.00	4.00	4.00	7.67	7.25	8.33	8.00	8.33

### Construct validity

Construct (external) validity was also analyzed by exploring the correlations of scores on the PSS-10, brief COPE-A and MTQ-10. The results of the correlation analysis are presented in Tables 3 and 4.

**Table 3:** Correlation of PSS-10 and COPE-A scores

	1	2	3	4	5	6	7	8	9
1. perceived helplessness	–								
2. perceived self-efficacy	0.38***	–							
3. Perceived stress (total)	0.81***	0.83***	–						
4. Socio-emotional support	0.20***	− 0.05*	0.08***	–					
5. Turning to religion	0.18***	0.07**	0.16***	0.21***	–				
6. Avoidance	0.55***	0.33***	0.51***	0.32***	0.26***	–			
7. Acceptance	0.11***	− 0.10***	0.00	0.25***	0.06**	0.21***	–		
8. Humor	− 0.02	− 0.16***	− 0.10***	0.22***	0.06**	0.17***	0.28***	–	
9. Problem-focused coping	− 0.04	− 0.35***	− 0.24***	0.33***	0.05*	0.01	0.35***	0.27***	–

*p < 0.05, **p < 0.01, ***p < 0.001

**Table 4:** Correlation scores for the PSS-10 and MTQ-10 scales

Variable	Perceived helplessness	Perceived self-efficacy	PSS_total	MTQ_total
1. Perceived helplessness	–			
2. Perceived self-efficacy	0.39***	–		
3. PSS_total	0.81***	0.83***	–	
4. MTQ_total	− 0.42***	− 0.47***	− 0.54***	–

*p < .05, ** p < .01, *** p < .001

As shown in Table 3, significant correlations were observed for all PSS-10 and COPE-A scales, excluding the correlation of the perceived helplessness subscale with problem-focused coping, and humor, as well as PSS total and acceptance. The magnitude of the correlations did not exceed 0.55 (for the perceived helplessness and avoidance subscales). Table 4 shows the significant negative correlations of perceived helplessness, perceived self-efficacy, PSS-10 total, and MTQ-10 scores.

### Sex invariance

Measurement invariance across sexes was evaluated to assess the applicability of the PSS-10 in both male and female groups (see Table 5). Model 1 was used to assess configural invariance and demonstrated a good fit to the data (CFI = 0.986, RMSEA = 0.074). Model 2 was used to estimate metric invariance and demonstrated a good fit to the data (ΔCFI = 0.001, ΔRMSEA = 0.002, p = 1.000), indicating that the data are sex invariant in terms of factor loadings. Model 3 was used to evaluate scalar invariance and showed a good fit to the data (ΔCFI = 0.001, ΔRMSEA = 0.001, p < 0.001). Thus, the null hypothesis was rejected because the intercepts of the items were equal across the sexes at p < 0.001.

**Table 5:** Model fit indices regarding measurement invariance across sexes.

	CFI	RMSEA	Baseline test			Difference test				
			χ^2^	df	p	│ΔCFI│	│ΔRMSEA│	Δχ^2^	Δdf	p
Model 1	0.986	0.074	765.597	102	< .001					
Model 2	0.985	0.072	828.582	118	< .001	0.001	0.002	62.985	6	1.000
Model 3	0.985	0.075	828.582	108	< .001	0.001	0.001	− 9.256e-8	10	< .001

## Discussion

The main goal of this study was to evaluate the psychometric properties of the Perceived Stress Scale in Russian-speaking adolescents. In this study, factor structure and reliability, external validity, and measurement invariance across sexes in terms of configural, metric and scalar invariance were assessed. Thus, the use of the Russian version of the PSS-10 in adolescents was confirmed.

Confirmatory factor analysis revealed an initial 2-factor structure, which demonstrated satisfactory model fit indices. The final version included 10 items and 2 subscales (perceived helplessness and perceived self-efficacy). The perceived helplessness subscale includes 6 items, and the perceived self-efficacy subscale consists of 4 items. Both scales have a high internal reliability, with Cronbach's alpha above 0.77. The results are consistent with other studies that have demonstrated a two-factor structure in adolescent samples, as shown by Kechter et al.^53^, Liu et al.^22^, and Whitney et al.^54^.

The study confirmed the construct validity of the PSS-10 by examining correlations of PSS-10 scores with scores on the COPE-A and MTQ-10 scales for adolescents. Positive correlations were found between the perceived self-efficacy subscale of the PSS-10 and the turning to religion and avoidance subscales of the COPE-A, while negative correlations of the perceived self-efficacy subscale with the humor, acceptance, socioemotional support and problem-focused coping subscales were observed. The positive relationship between the avoidance subscale and the perceived self-efficacy subscale may be explained by maladaptive coping behavior, which reflects the inability to manage stress. Although the size of the correlations was modest, similar results were reported in earlier study of adolescent groups^55^. Negative correlations were found for the perceived helplessness, perceived self-efficacy and total PSS-10 scores with scores on the mental toughness scale. Negative correlations of perceived stress and mental toughness have been demonstrated previously^56^. A negative relationship between stress and mental toughness was found, which indicates that mental toughness is an adaptation to stress, with low stress scores observed in individuals with high mental toughness^57^.

The measurement invariance of the PSS-10 was initially evaluated in a sample of Russian-speaking adolescents to determine the suitability of using the scale in both sexes. Three models were assessed: configural, metric, and scalar invariance. Configural and metric invariance were supported, which is consistent with findings from studies in other countries^22,58–61^. The configural invariance of the model demonstrates that the same constructs are measured by the PSS-10 in both sexes. Metric invariance indicates that the factor loadings are similar between males and females. The significance of the fit indices in the scalar invariance model indicates variance in the intercepts. However, it can be concluded that the PSS-10 can be used in both sexes due to the configural invariance, which indicates the unity of the construct.

The development of the PSS-10 for use in diagnosing stress in adolescents is important, as there is a lack of such tools available in Russia. This instrument has wide-ranging practical applications, and it is recommended for use by school psychological services, researchers, and administrators. The PSS-10 can be utilized to assess acute stress reactions resulting from communication problems in children's groups, bullying, and increased workloads in high school. The diagnostic results can be used to develop individual recommendations and training programs that teach effective coping strategies to adolescents, ultimately reducing the negative consequences of stress.

Moreover, the PSS-10 can be widely used in research due to its several advantages. First, it enables the comparison of perceived stress levels between adolescents and adults. Second, it can be used in various countries, allowing for cross-cultural analysis of perceived stress. Finally, its brevity makes it a time-saving tool, especially for large-scale testing.

The developed tool can be used in various groups in the future, including oncological patients. It is well known that cancer patients may experience negative psychological and psychophysiological symptoms such as increased stress levels, anxiety, depression, sleep disturbances, and reduced mental health. To alleviate stress, various coping strategies can be utilized, including behavioral and cognitive strategies that help to reduce the pressure of a stressful situation. However, different coping strategies have varying degrees of effectiveness. Therefore, assessing the level of stress and other psychological reactions is critical in developing effective measures for management. However, the diagnostic methods used by psychologists for these purposes are often based on outdated norms and need to be standardized. The PSS-10 has satisfactory psychometric properties and can be used for diagnostic purposes.

It is important to consider that stress can lead to serious disorders such as depression and posttraumatic stress disorder^62^. Timely diagnosis and the development of effective coping behavior can reduce the risk of developing these mental disorders, making the PSS-10 useful for professionals in the mental health field.

This study has an important limitation related to the age of the sample, which included only adolescents aged 13 and above. Further research is needed to validate and adapt the Perceived Stress Scale for use with younger adolescents.

Another limitation of the study may be that the type of stressor affecting adolescents was not assessed during the measurement. The type of stressor can be an important diagnostic criterion^63^, determining the response to stress. Moreover, the COVID-19 pandemic is a source of various types of stressors that affect the response of adolescents to stress in different ways^64^. In future research, it is important to assess the response to a specific type of stressor in adolescents in relevant situations.

The study confirms that the PSS-10 has satisfactory psychometric properties and can be used for stress assessment in adolescents. Its brevity makes it suitable for quick diagnostics and attractive to psychologists working with large groups of adolescents, such as those in schools. The questionnaire can be useful in various areas, including health care and academic environments where bullying may occur.

## Methods

### Participants

Participants in the study were 4834 adolescents between the ages of 13 and 17 years. Technical outliers were removed from the dataset during preparation. These outliers were identified and excluded based on the following criteria:

- similarity of answers (the same answer was selected by the respondent for all items),
- item response duration (less than 1.5 s),
- total questionnaire duration (less than 400 s),
- variability of item response duration (standard deviation of the item response duration less than 6 s or more than 60 s).

Regarding the item duration response, the left peak of the bimodal distribution was removed, as was the right peak for the total questionnaire duration. All observations identified under at least one of the above criteria were excluded. Subsequently, the Mahalanobis distance of the item response duration was assessed. Observations exceeding the 0.90 quantile were excluded. The final sample consisted of 3530 adolescents in grades 6–11 with a mean age of 15.3 years (SD = 1.05 years). The sample included 2192 girls, 1276 boys, and 62 participants who did not indicate their sex. The age composition of the sample included 13-year-olds (n = 65), 14-year-olds (n = 864), 15-year-olds (n = 1136), 16-year-olds (n = 933), and 17-year-olds (n = 532).

### Procedure

The data were collected online using personal computers as a part of the study on stress and coping behavior in adolescents during the COVID-19 pandemic. Students were also asked about their sex, age, and socioeconomic status.

### Ethics approval

The study was conducted according to the guidelines of the Declaration of Helsinki and was approved by the Ethics Committee of the Psychological Institute of the Russian Academy of Education.

### Informed consent statement

The informed consent of all adolescents' parents was obtained.

### Scales

#### Perceived Stress Scale (PSS-10)

The 10-item Perceived Stress Scale was developed by Cohen (1988)^48^ to measure the level of perceived stress. The scale consists of 10 questions, and in this study, the Russian version of the questionnaire^52^ was used. Translation and back translation of the questionnaire was provided in the study of Ababkov and colleagues^52^. Authors reported that translation and back translation were performed by professional translators. The Russian version also has 10 questions and a two-factor structure: perceived helplessness and perceived self-efficacy. The total score of the questionnaire is calculated by adding the scores of each item. Respondents are asked to indicate how often they experienced a certain condition in the last month using a Likert scale that ranged from 1 ("never") to 5 ("often"). Items 1, 2, 3, 6, 9, and 10 are part of the perceived helplessness subscale. Each item is rated on a scale of 1 to 5 points, and the scores are summed. Items 4, 5, 7, and 8 are part of the perceived self-efficacy subscale. Each item is rated on a scale from 1 to 5 points, but the scores on this subscale are reversed, such that 1 = 5, 2 = 4, 3 = 3, 4 = 2, and 5 = 1. The scores are then summed. To calculate the total score of the Perceived Stress Scale, the scores of all 10 questions (items) are summed. The scores of both subscales (perceived helplessness and perceived self-efficacy) can also be summed to obtain the total score of the questionnaire.

#### The brief version of the Coping Orientation for Problem Experiences for adolescents (Brief-COPE-A; 29 items)

In our study, we used the Brief-COPE-A version developed by Marakshina et al.^26^ for Russian adolescents. This version was based on the Russian version of the COPE^65^ using the translation by Rasskazova et al. The original Coping Orientation for Problem Experiences (COPE) questionnaire was developed by Carver et al.^66^. The Brief-COPE-A version was used to assess the external validity of the PSS-10 and is intended to diagnose coping strategies in adolescents aged 13–17. The questionnaire includes 29 items that assess six coping strategies: problem-focused coping, avoidance, socioemotional support, acceptance, turning to religion, and humor. Items with opposite meanings were reverse coded, with 1 = 4, 2 = 3, 3 = 2, and 4 = 1. The total score for each coping strategy was calculated as the average of all scores on that strategy.

#### Mental toughness questionnaire (MTQ-10)

The MTQ-10 is a brief version of the MTQ-48. The original version of the questionnaire was developed in 2002^67^. The questionnaire is used to assess mental stability, especially in sports psychology. The original version includes 48 questions; brief versions have also been developed consisting of 18 and 10 items^68^. The 48-item version evaluates the 4 main components of psychological toughness (control, challenge, determination, and confidence)^69^. The 10-item version contains one scale^70^. The general component of psychological resilience reflects individual resilience and the ability to achieve success. Responses are indicated on a Likert scale from 1 (“strongly disagree) to 5 (“strongly agree”). The answers to questions 1, 4, 5, 8, 9, and 10 were coded on a scale from 1 to 5. Items 2, 3, 6, and 7 were reversed scored, with 1 = 5, 2 = 4, 4 = 2, and 5 = 1. Mental toughness was recorded as the sum of all scores on ten items. The MTQ-10 was used to assess the external validity of the PSS-10. Cronbach’s alpha in our sample of adolescents was 0.73.

### Data analysis

Data analysis was performed in R version 4.1.2 (including dataset preparation, sampling procedure, reliability calculation, correlation analysis) and JASP 0.16.1.0 (confirmatory factor analysis, descriptive statistics, correlation analysis, reliability calculation, structural equation modeling, SEM for measurement invariance). Spearman's correlation analyses were conducted. Measurement invariance across sexes was evaluated using configural invariance, metric invariance, and scalar invariance.

Initially, skewness and kurtosis were calculated: for Total Perceived Stress Scale skewness was 0.160 and kurtosis was − 0.02. Values smaller than 1 indicated normal distribution of the data.

Our theoretical assumptions, based on a previous adaptation of the Perceived Stress Scale (PSS) in adults, included a two-factor model. We used confirmatory factor analysis to confirm the factorial structure of the questionnaire, and we evaluated the fit of the model data using fit indices such as values of the standardized root mean squared residual (SRMR) of < 0.08, Tucker–Lewis index (TLI) scores that approach 1, comparative fit index (CFI) > 0.95, and root mean square error of approximation (RMSEA) < 0.08^71–74^. We also evaluated configural, metric, and scalar invariance using the DWLS estimator in SEM. Fit indices such as ΔCFI and ΔRMSEA were assessed. Configural invariance was assessed by CFI and RMSEA values. We considered ΔCFI < 0.010 and ΔRMSEA < 0.015 to demonstrate metric and scalar invariance^75,76^.

To assess the reliability (internal consistency) of the scale, we used Cronbach's alpha. We considered a Cronbach's alpha value of 0.7 or higher to confirm the reliability of the scale^77^.

### Supplementary Information

Supplementary Information.

## Data Availability

The datasets used and analysed during the current study available from the corresponding author on reasonable request.
