# Supplementary material for: Factor structure and psychometric properties of the Perceived Stress Scale in Russian adolescents
Source: Sci Rep. 2024 Jan 8;14:775. doi: 10.1038/s41598-023-51104-1 (PMC10774267; doi:10.1038/s41598-023-51104-1)
Supplement: Supplementary file 1 — Supplementary Information. [file 41598_2023_51104_MOESM1_ESM.docx]

**Supplementary Information**

**Perceived Stress Scale in English and Russian**

*Instruction for participants*

Read the statements carefully and evaluate how often over the past month:

| № of item | English | Russian |
| --- | --- | --- |
| 1 | How often have you been upset because of something that happened unexpectedly? | Ты испытывал(-а) беспокойство из-за непредвиденных событий |
| 2 | How often have you felt that you were unable to control the important things in your life? | Тебе было сложно контролировать важные события твоей жизни |
| 3 | How often have you felt nervous and "stressed"? | Ты испытывал(-а) нервное напряжение или стресс |
| 4 | How often have you felt confident about your ability to handle your personal problems? | Ты чувствовал(-а) уверенность в том, что справляешься с решением своих личных проблем |
| 5 | How often have you felt that things were going your way? | Чувствовал(-а), что все идет так, как тебе хочется |
| 6 | How often have you found that you could not cope with all the things that you had to do? | Думал(-а), что не можешь справиться с тем, что тебе нужно сделать |
| 7 | How often have you been able to control irritations in your life? | Ты мог(-ла) справиться со своей раздражительностью |
| 8 | How often have you felt that you were on top of things? | Чувствовал(-а), что владеешь ситуацией |
| 9 | How often have you been angered because of things that happened that were outside of your control? | Чувствовал(-а) раздражение из-за того, что происходящие события выходили из-под твоего контроля |
| 10 | How often have you felt difficulties were piling up so high that you could not overcome them? | Тебе казалось, что накопившиеся трудности достигли такого предела, что ты не мог(-ла) их контролировать |

*Response scale*: 1 - Never 2 – Almost never 3 - Sometimes 4 – Fairly often 5 - Often
